# Supplementary material for: Universal neural networks for real-time earthquake early warning trained with generalized earthquakes
Source: Commun Earth Environ. 2024 Sep 27;5(1):528. doi: 10.1038/s43247-024-01718-8 (PMC11488472; doi:10.1038/s43247-024-01718-8)
Supplement: Supplementary file 2 — Supplementary Materials [file 43247_2024_1718_MOESM2_ESM.pdf]

Supplementary Materials for

Universal Neural Networks for Real-Time Earthquake Early Warning Trained with Generalized Earthquakes

Xiong Zhang*, Miao Zhang

*Corresponding author. Email: zxiong@mail.ustc.edu.cn

This PDF file includes:

Supplementary Figures 1 to 5
Supplementary Table 1

Other Supplementary Materials for this manuscript include the following:

Supplementary Movies 1 to 2

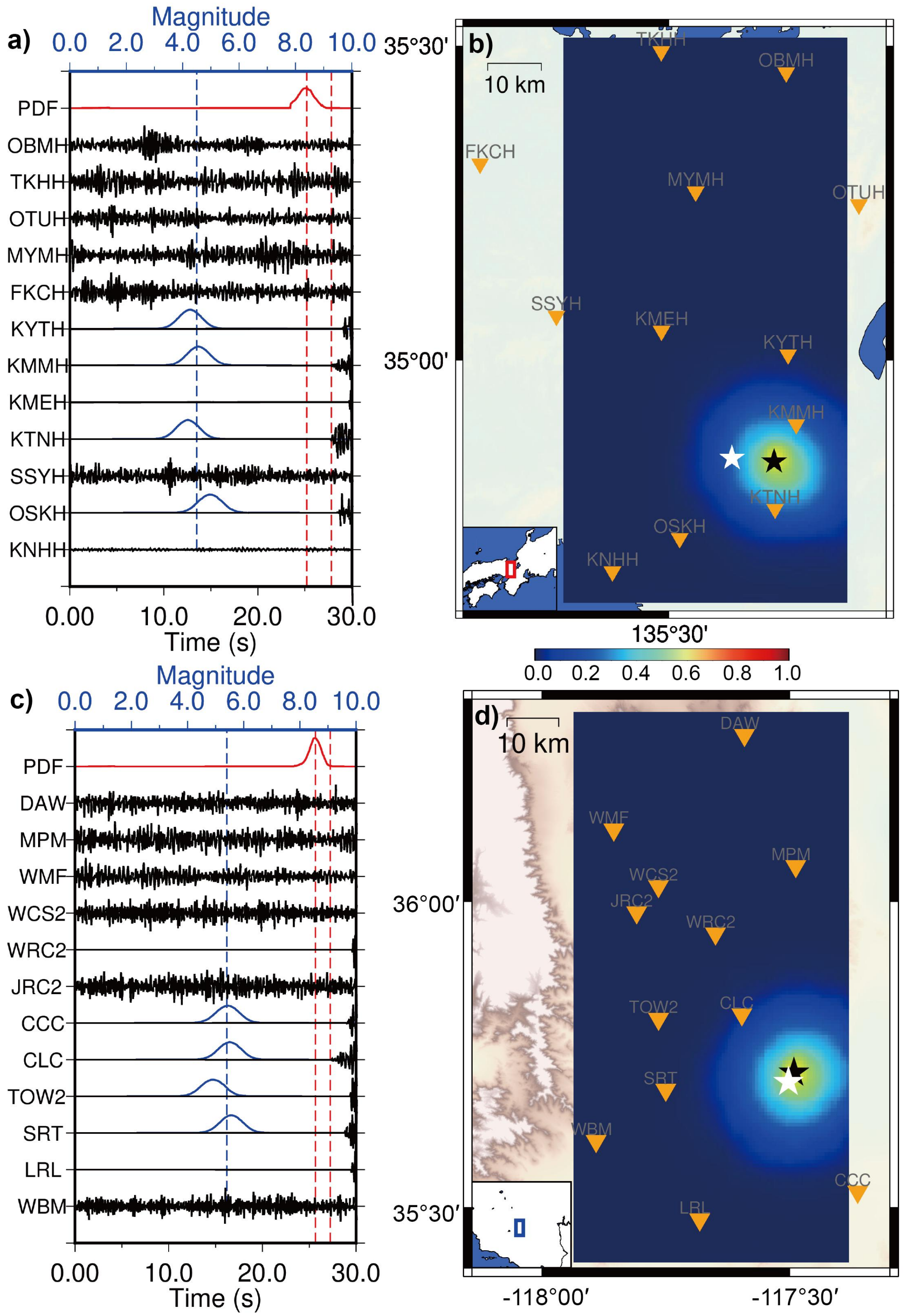

Supplementary Figure 1. The snapshots for the first alerts. Panels a and b are the results for the main shocks on June 18, 2018, in Osaka, Japan. Panels c and d are the results for the main shocks on July 4, 2019, in Ridgecrest, US. The predicted P arrivals of the first triggered stations are slightly earlier than the picked onsets (red dashed lines).

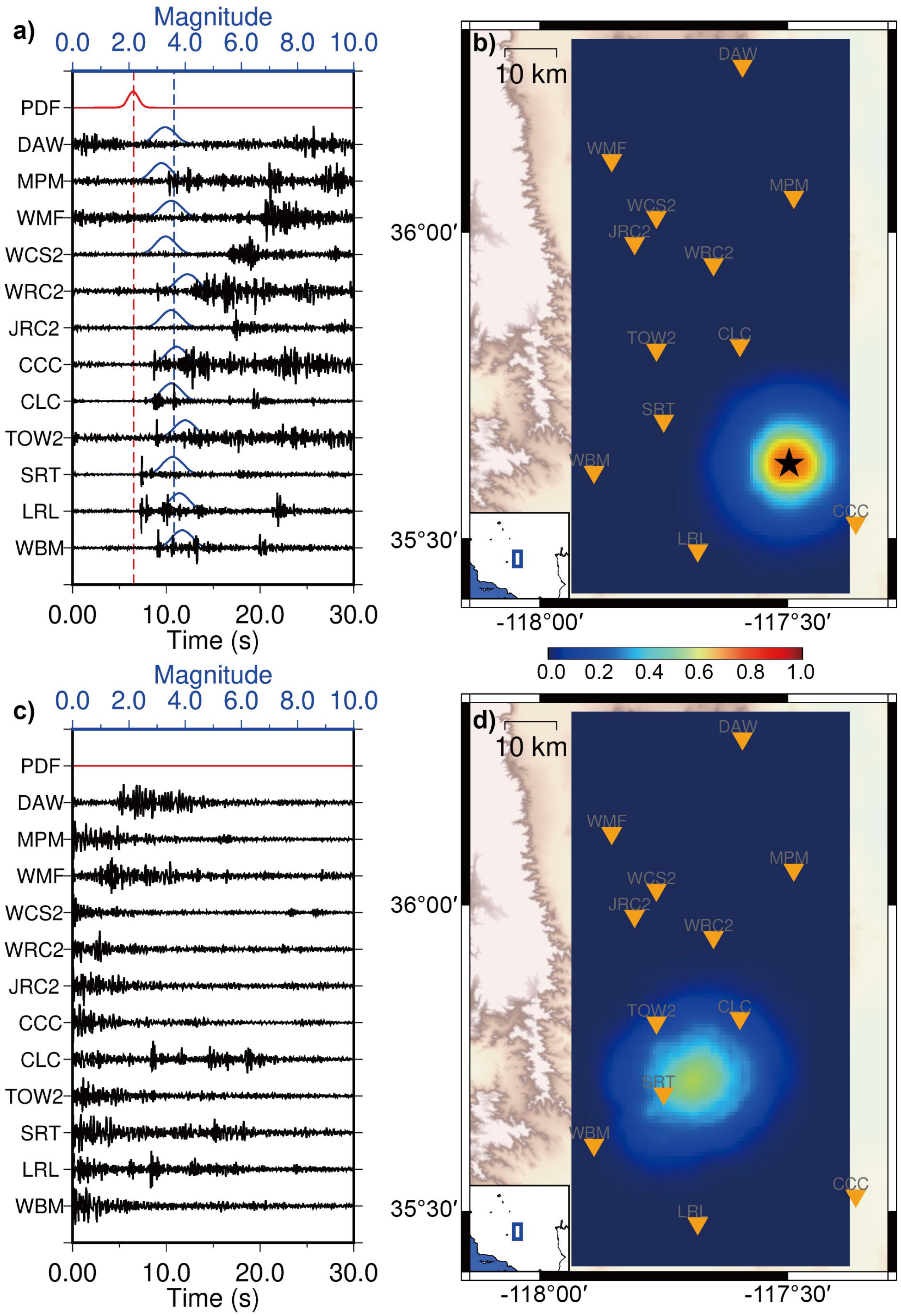

Supplementary Figure 2. The interfered earthquakes in the truncated time windows. The detected and located earthquake although the waveforms are contaminated by another earthquake (**a**, **b**); the predicted magnitude is M 3.6; the detection and location PDFs are 0.89 and 0.75, respectively. The missed earthquake contaminated by the coda waves from a relatively large earthquake (**c**, **d**).

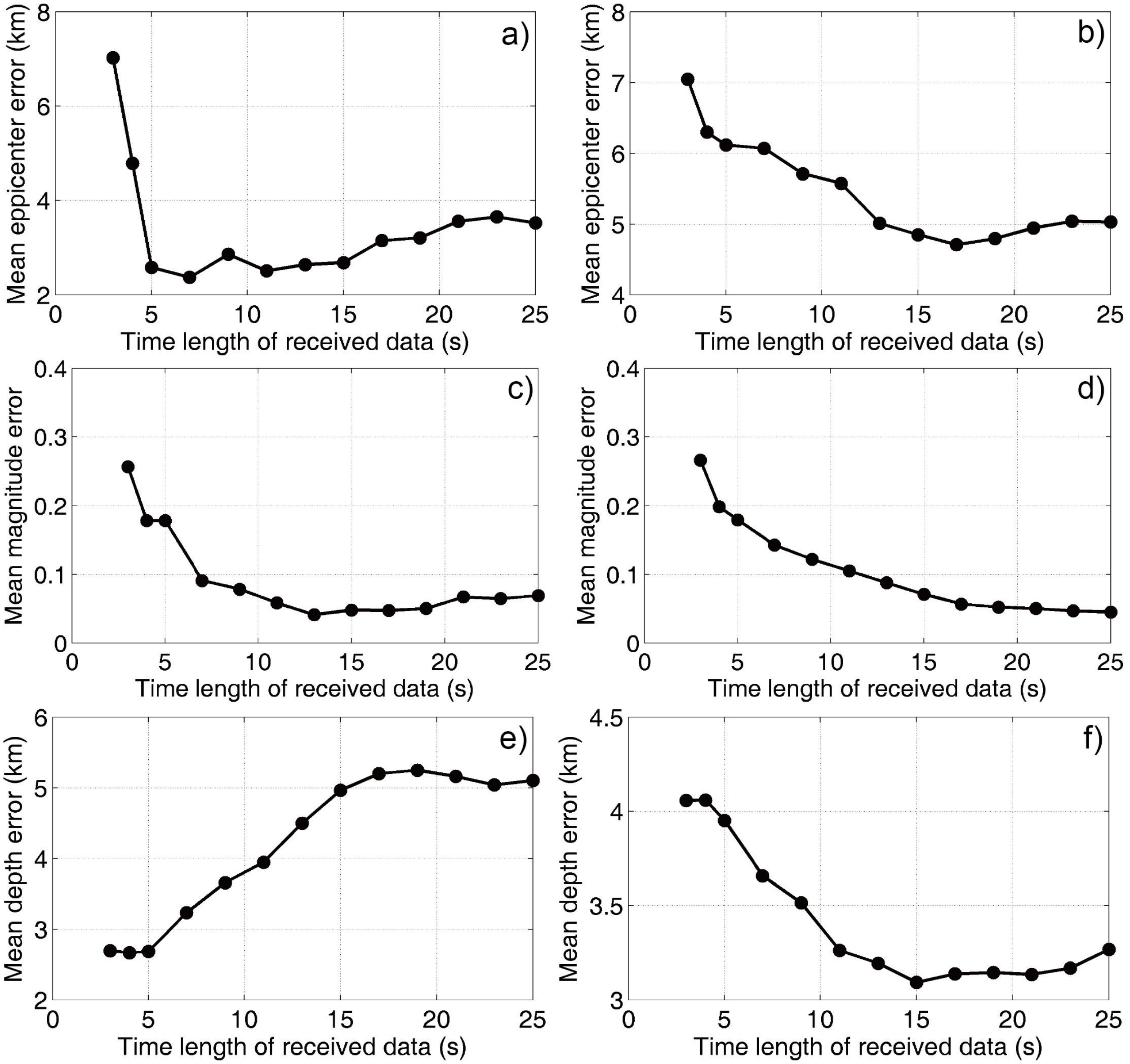

Supplementary Figure 3. Parameter error analysis. Panels **a**, **c**, and **e** are the results for the 179 earthquakes in Osaka, Japan. Panels **b**, **d**, and **f** are the results for the 349 earthquakes in Ridgecrest, US. The input time windows contain different lengths of effective signals for neural network testing, and the errors are calculated between the predicted and cataloged results.

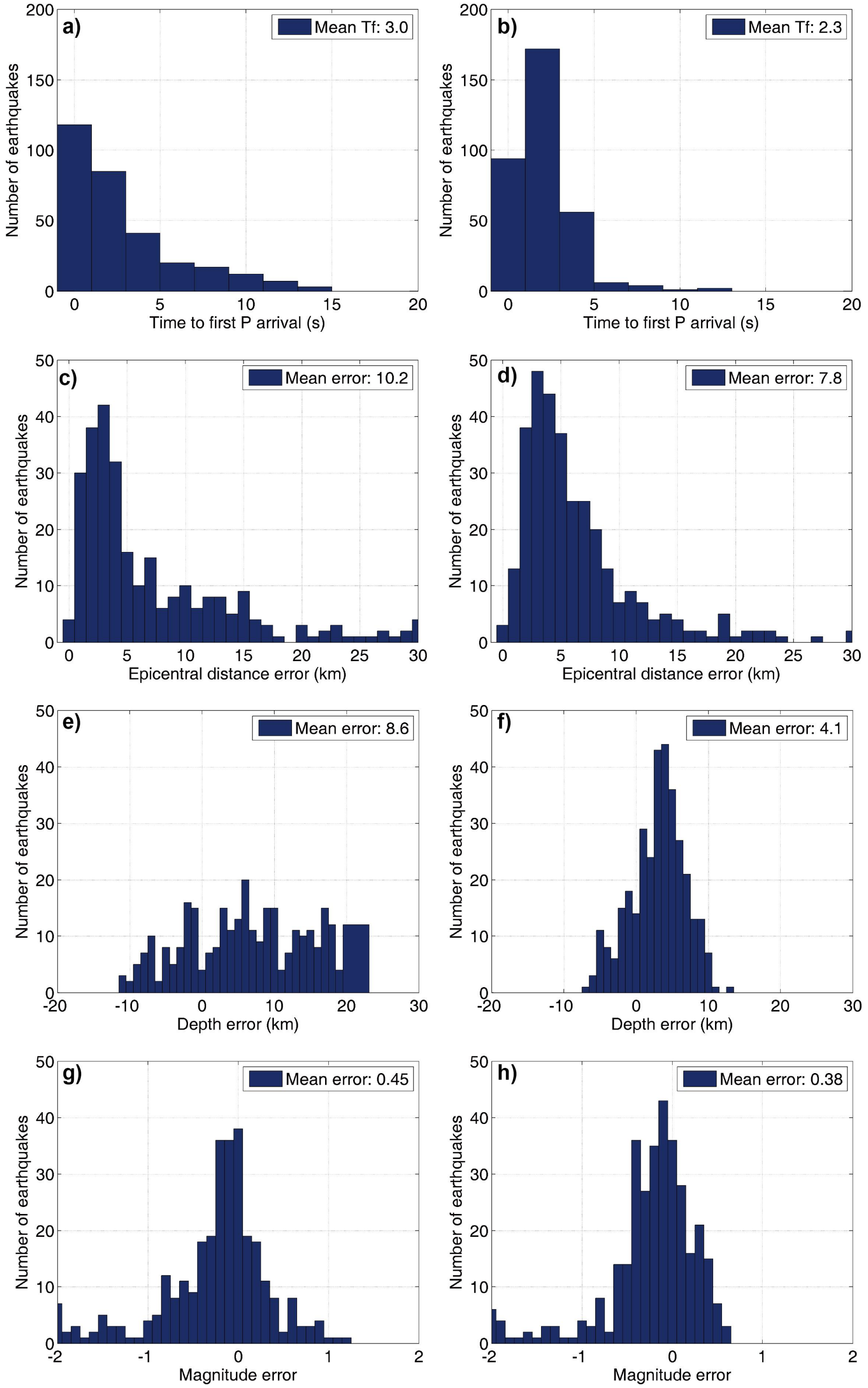

Supplementary Figure 4. The comparison between the traditional picking-based method and the neural network method. Panels **a**, **c**, **e**, and **g** are the statistic results of first alarm time, epicentral distance errors, depth errors, and magnitude errors for traditional method. Panels **b**, **d**, **f**, and **h** are the corresponding statistic results for neural network method.

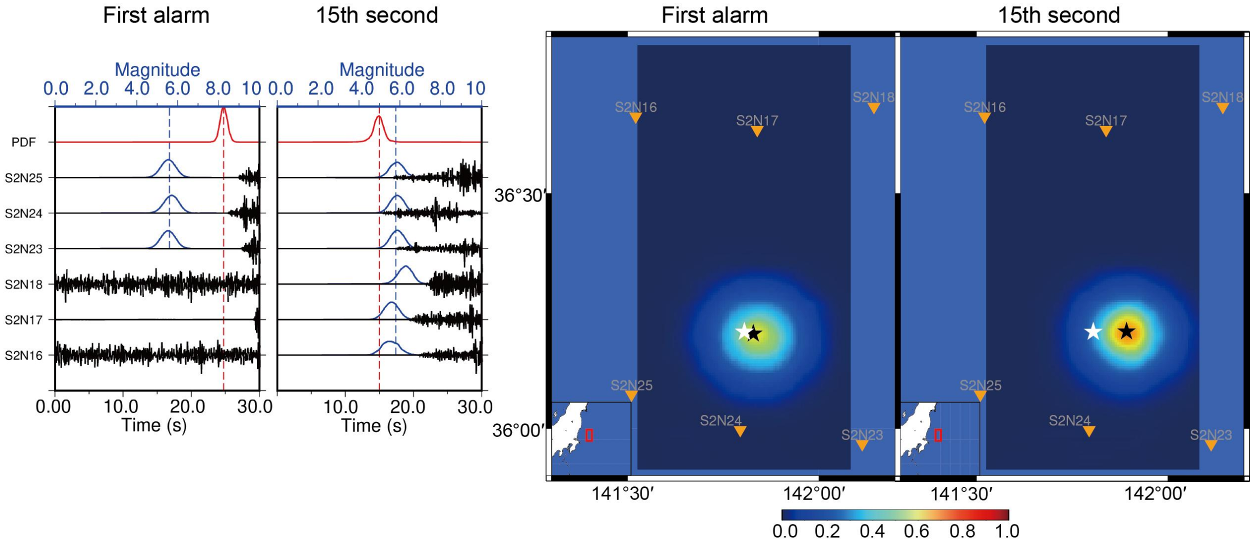

Supplementary Figure 5. The testing results for the M 6.0 offshore Ibaraki earthquake in Japan on August 4, 2021. The monitoring snapshots at first alarm and 15th second from first P arrival are shown in the figure. The black, blue, and red curves represent the input waveforms (Z components), magnitude PDFs, and detection PDFs, respectively; the blue dashed lines mark the mean of the predicted magnitudes; the black and white stars in the right figures are the predicted and cataloged earthquake locations. The first alarm time is 5.2 seconds after the onset of initial P arrival, with epicentral location, depth, and magnitude errors of 2.1 km, 12.3 km, and 0.4, respectively. By the 15th second, the magnitude error improves to 0.2, though the epicentral location error increases to 8.4 km. The relatively large location errors may result from complex velocity structures, as most training samples for the location network are from onshore earthquakes.

Supplementary Table 1. The parameter settings for traditional picking-base method and neural network method.

<table>
<tr><th colspan="2">Traditional picking-based method</th><th>Neural network</th></tr>
<tr><td>Triggers</td><td>The LTA/STA time windows are set to 2s and 1s.
The threshold value of the LTA/STA is 20.0.
The signal to noise ratio of the waveform is above 4.0.
The $log(P_d)$ values are in the range from -5.5 to 3.5.</td><td rowspan="4">Detection and location PDFs are above 0.7 and 0.6.
The effective signals are longer than 2 seconds and maximum PDF exceeds 0.6 for magnitude estimation.</td></tr>
<tr><td>Location</td><td>The grid sizes are 5 km and 4 km in horizontal and depth directions for grid search method.
The arrival time errors at 4 stations are less than 1 second.</td></tr>
<tr><td>Magnitude</td><td>The magnitude is computed by empirical formula:
$M=1.04log(P_d)+1.27log(R)+5.16$.
The maximum ampltidues of the waveforms within 4 seconds are used to estimate the magnitude.</td></tr>
<tr><td>Alert criteria</td><td>At least 4 stations triggered.</td></tr>
</table>
