# Supplementary material for: Universal neural networks for real-time earthquake early warning trained with generalized earthquakes
Source: Commun Earth Environ. 2024 Sep 27;5(1):528. doi: 10.1038/s43247-024-01718-8 (PMC11488472; doi:10.1038/s43247-024-01718-8)
Supplement: Supplementary file 3 — Description of Additional Supplementary Files [file 43247_2024_1718_MOESM3_ESM.pdf]

Description of Additional Supplementary Files

File name: Supplementary Movie 1

Description: Real-time monitoring of one hour continuous data from 07:58:00 to 08:57:59 including the MJMA 6.1 earthquake in Osaka, Japan. Left panel: truncated continuous waveforms for early warning (30 s) and the detection and magnitude PDFs. Time is relative to the Twin. Right panel: location probability distribution in map view. Event locations are documented by white circles (scaled by their magnitude) along with their origin time (Torg) when their location and detection PDFs meet the threshold settings.

File name: : Supplementary Movie 2

Description: Real-time monitoring of one hour continuous data from 17:00:00 to 17:59:59 including the Mw 6.4 earthquake in Ridgecrest, US. Left panel: truncated continuous waveforms for early warning (30 s) and the detection and magnitude PDFs. Time is relative to the Twin. Right panel: location probability distribution in map view. Event locations are documented by white circles (scaled by their magnitude) along with their origin time (Torg) when their location and detection PDFs meet the threshold settings.
